# Necrotic cells influence migration and invasion of glioblastoma via NF-κB/AP-1-mediated IL-8 regulation

**DOI:** 10.1038/srep24552

**Published:** 2016-04-14

**Authors:** So-Hee Ahn, Hyunju Park, Young-Ho Ahn, Sewha Kim, Min-Sun Cho, Jihee Lee Kang, Youn-Hee Choi

**Affiliations:** 1 Department of Physiology, Ewha Womans University School of Medicine, Seoul 911-1, Korea; 2 Tissue Injury Defense Research Center , School of Medicine, Ewha Womans University, Seoul, Korea; 3 Department of Molecular Medicine , School of Medicine, Ewha Womans University, Seoul, Korea; 4 Department of Pathology, School of Medicine, Ewha Womans University, Seoul, Korea

## Abstract

Glioblastoma multiforme (GBM) is the most common primary intracranial tumor in adults and has poor prognosis. Diffuse infiltration into normal brain parenchyma, rapid growth, and the presence of necrosis are remarkable hallmarks of GBM. However, the effect of necrotic cells on GBM growth and metastasis is poorly understood at present. In this study, we examined the biological significance of necrotic tissues by exploring the molecular mechanisms underlying the signaling network between necrotic tissues and GBM cells. The migration and invasion of the GBM cell line CRT-MG was significantly enhanced by treatment with necrotic cells, as shown by assays for scratch wound healing and spheroid invasion. Incubation with necrotic cells induced IL-8 secretion in CRT-MG cells in a dose-dependent manner. In human GBM tissues, IL-8 positive cells were mainly distributed in the perinecrotic region, as seen in immunohistochemistry and immunofluorescence analysis. Necrotic cells induced NF-κB and AP-1 activation and their binding to the IL-8 promoter, leading to enhanced IL-8 production and secretion in GBM cells. Our data demonstrate that when GBM cells are exposed to and stimulated by necrotic cells, the migration and invasion of GBM cells are enhanced and facilitated via NF-κB/AP-1 mediated IL-8 upregulation.

Astrocytoma is one of the most common brain tumors in humans. Grade IV astrocytoma, also called glioblastoma multiforme (GBM), is considered the most malignant glial tumor^1^. The remarkable features of GBM include local invasion, diffuse infiltration into adjacent brain tissue and the presence of necrosis^2^. Despite optimal treatments, patients with GBM have a poor prognosis with a 5-year survival rate of 5% due to diffuse infiltration into normal brain parenchyma and rapid growth^3^. Migration and proliferation of GBM are influenced by several pathogenic factors, including glioblastoma stem cells and various signaling pathways initiated by cytokines and chemokines^4^,^5^,^6^. Particularly, IL-8 is thought to be one potential mediator of GBM malignancy and pathogenesis.

Interleukin-8 (IL-8, CXCL8) is one of the CXC chemokines, which plays multiple roles in immune response and cancer. IL-8 is produced by various types of cells, including macrophages, epithelial cells, airway smooth muscle cells, and endothelial cells^7^. IL-8 is a neutrophil chemotactic factor and acts as an important mediator of the innate immune response^8^,^9^. Furthermore, IL-8 contributes to a more invasive phenotype in a variety of cancers, including breast, ovarian, pancreatic, thyroid, and glioblastoma, by promoting tumoral angiogenesis and metastasis^10^,^11^,^12^,^13^,^14^. Aberrant increase of IL-8 occurs in response to lipopolysaccharide (LPS), inflammatory cytokines such as TNF-α and IL-1, death receptor activation, and various cellular stressors including ischemia and hypoxia^7^,^15^.

Necrosis is a characteristic feature of advanced solid tumors, caused by ischemia and hypoxia^16^,^17^. In GBM, necrosis is a key diagnostic feature. Histologically, the presence of necrosis upgrades a malignant astrocytoma (grade III) to GBM (grade IV), which is the most severe tumor grade^1^,^2^. Several clinical studies demonstrate that the presence of biological necrosis has a negative overall impact on survival and is a poor prognostic factor^18^. However, the reason that increased necrosis is associated with decreased survival rate and contributes to poor prognosis is not clearly understood.

Due to the biological significance of necrosis in GBM, many studies have addressed the molecular mechanisms of the development of necrosis; however, little is known about the biological functions of necrotic tissue in GBM. In this study, we investigated the effect of necrosis on GBM migration and invasion in the human glioblastoma cell line, CRT-MG. We demonstrate that necrotic cells not only induce the expression of the CXC chemokine IL-8, but also promote migration and invasion of human glioblastoma cells. These responses were dependent on necrotic cell-induced activation of NF-κB and AP-1 signaling pathways. To our knowledge, this is the first report to address the effect of necrotic cell/necrosis on the migration and invasion of human glioblastoma cells. These findings support the notion that necrotic tissues may play a role in tumor cell migration and invasion by activating intratumoral signaling pathways and inducing chemokine expression in glioblastoma.

## Results

### Necrotic cells induce migration of glioblastoma cells

**Figure 1:**
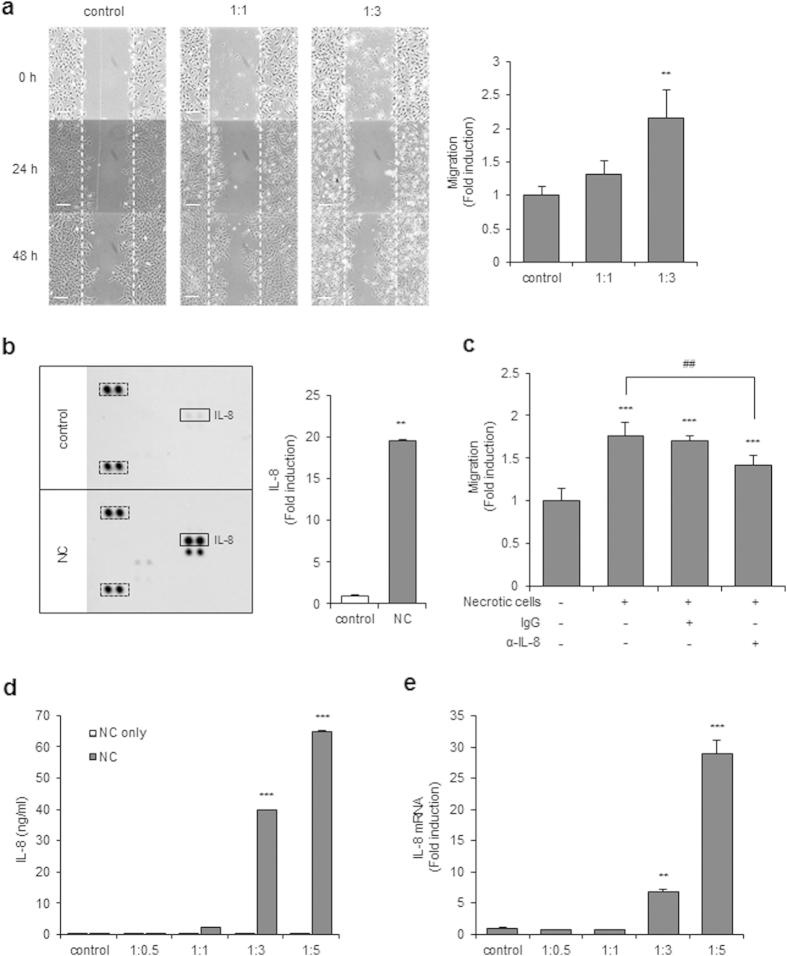
Necrotic cells increase migration of CRT-MG cells. (**a**) Representative micrographs of the scratch wound healing assay in CRT-MG cells treated with necrotic CRT-MG cells incubated for 0, 24, and 48 h. scale bar = 100 μm. Migration activity was measured by calculating the area that advanced from boundary lines of scratch to cell-free space for 48 h. Data are presented as the fold induction compared with each untreated control cells (right). **P < 0.01 vs. control. (**b**) Cell lysates of CRT-MG cells treated or untreated with necrotic cells for 24 h were analyzed using a chemokine array. The duplicate spots corresponding to IL-8 are indicated by a solid box (left), and reference spots positive control are indicated by a dashed line box. The intensity of spots corresponding to IL-8 and positive control was quantified using Image J software and subtracted from the background, then expressed as a ratio to the positive control. The value of IL-8 in the untreated control was set as 1. **P < 0.01 vs. control. NC, necrotic cells. (**c**) The scratch wound healing assay was performed using CRT-MG cells treated with necrotic CRT-MG cells in the presence or absence of either anti-IL-8 neutralizing antibody (2.5 μg/ml) or control nonspecific goat-IgG (2.5 μg/ml). Data shown are representative of at least three experiments. ***P < 0.001 vs. control, ^*##*^*P* < 0.01 vs. necrotic cells. (**d**) CRT-MG cells treated with different ratios of necrotic cells (NC) or necrotic cells only (NC only) for 24 h as indicated. After incubation, supernatants from each condition were collected, and IL-8 protein levels were measured by ELISA. ***P < 0.001 vs. control. (**e**) Quantitative real-time PCR (qRT-PCR) analysis was performed for CRT-MG cells incubated in the absence or presence of necrotic CRT-MG cells for 24 h. Data were presented as fold induction compared with control cells. **P < 0.01, ***P < 0.001 vs. control.

To test whether necrotic tissues affect the migration activity of GBM, CRT-MG, U251-MG and U87-MG cells were treated with necrotic CRT-MG, U251-MG and U87-MG cells respectively, and cell migration was assessed with a scratch wound healing assay. Preparation of the necrotic cells is described in the Methods section and the quantitation of necrosis was performed by flow cytometry (Supplementary Fig. S1). The extent of migration of CRT-MG, U251-MG and U87-MG cells was significantly increased in the presence of necrotic CRT-MG cells in a ratio-dependent manner (Fig. 1a and Supplementary Fig. S2a,b). Since several chemokines are reported to control the migration and invasion of cancer cells^19^, we next performed a chemokine array with the culture media from CRT-MG cells treated with necrotic cells. The chemokine array showed that secretion of several chemokines, including IL-8, was enhanced in necrotic cell-treated CRT-MG cells (Fig. 1b). To examine whether enhanced IL-8 directly mediated the migration of CRT-MG cells induced by necrotic cells, cells were treated with an anti-IL-8 neutralizing antibody or non-specific IgG in the presence of necrotic cells, and migration of CRT-MG cells was analyzed with the scratch wound healing assay (Fig. 1c). Co-treatment with IL-8 neutralizing antibody, but not IgG, significantly decreased the migration of CRT-MG cells. To determine whether necrotic cells directly induced IL-8 secretion in glioblastoma cells, CRT-MG cells were incubated with different ratios of necrotic CRT-MG cells for 24 h, and IL-8 protein levels in culture media were analyzed by ELISA, as described in the Methods section. Necrotic cells induced a marked increase in IL-8 protein levels in CRT-MG cells in a ratio-dependent manner; however, the necrotic cells did not release IL-8 (Fig. 1d). Similar effects of necrotic cells on IL-8 induction were also observed in U251-MG and U87-MG cells (Supplementary Fig. S2d,e). Expression of IL-8 mRNA was also drastically increased in necrotic cell-treated CRT-MG cells (Fig. 1e). These results clearly indicate that necrotic cells induce secretion of IL-8 and migration of CRT-MG glioblastoma cells.

### Strong IL-8 expression of perinecrotic tumor cells in human GBM tissue

**Figure 2:**
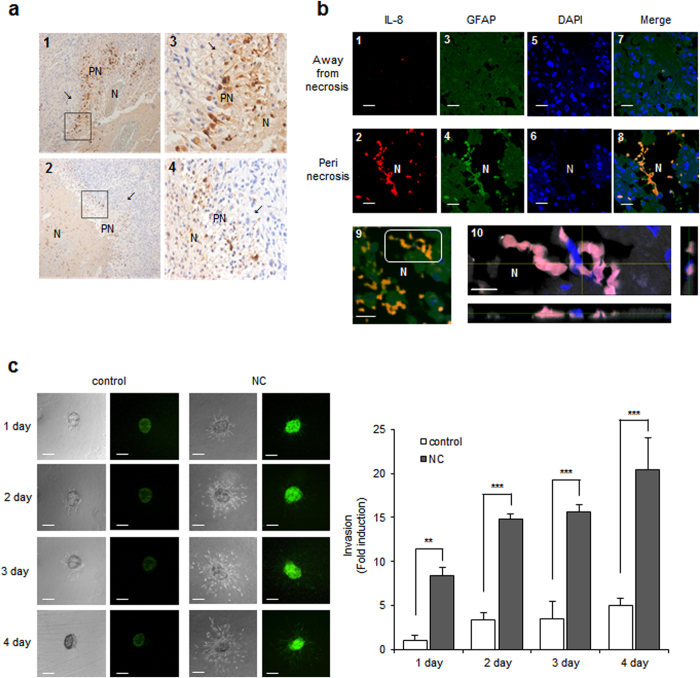
Necrotic cells induce IL-8 expression and cell invasion. (**a**) Immunohistochemical-l staining for IL-8 protein expression in the perinecrotic region was performed for 12 cases of the grade IV glioblastoma. Boxed areas of the necrotic border (1, 2, original magnification, ×100) are shown enlarged in the insets (3, 4, original magnification, ×400). The tumor cells distant from necrosis were IL-8 negative (arrow). PN, perinecrotic area; N, necrosis. (**b**) Double staining of IL-8 and GFAP in glioblastoma tissues. Tissues were immunostained with primary antibodies against IL-8 and the astrocyte marker GFAP. Nuclei were stained with DAPI. The experiment was repeated at least three times, with similar results (scale bar = 20 μm). Boxed areas of necrotic border are shown as a Z-stack projection image. YZ and XZ cross-sections are presented in the right and bottom panels (IL-8, red; GFAP, gray; Merged, pink) through the merged XY image showings the position of XZ or YZ cross-sections (pink) (10; scale bar = 10 μm; N, necrosis). (**c**) Invasion of CRT-MG cell clones stably transfected with construct IL-8p-d2EGFP was analyzed with a spheroid invasion assay. Spheroids were grown in the presence or absence of necrotic cells for up to 4 days and imaged by microscopy. Cell invasion was quantified by calculating the invading area as indicated in the Methods (right). The extent of invasion in the untreated control was set as 1 and data presented as the fold induction compared with untreated control cells. **P < 0.01, ***P < 0.001 vs. control. NC, necrotic cells. Data represent of three independent experiments. Scale bar represents 200 μm for all panels.

To explore whether necrotic tissues influence IL-8 expression levels in glioblastoma cells, we performed immunohistochemical staining (IHC) for IL-8 using human GBM tissues. Nine of the 12 samples tested showed positive cytoplasmic IL-8 staining of perinecrotic tumor cells. Positively stained cells were distributed along the necrotic border. Six cases revealed many positive cells forming a perinecrotic linear band-like pattern (Fig. 2a, upper), and three cases showed a few scattered positive cells around the necrotic region (Fig. 2a, lower). We additionally employed immunofluorescence to detect IL-8 expression in human GBM tissues. Samples were incubated with antibodies against IL-8 and the astrocyte marker GFAP. Strong IL-8 immunoreactivity was observed along the necrotic border, consistent with the IHC data (Fig. 2b2, red). In contrast, IL-8 was barely detectable in tumor cells located in areas away from necrosis (Fig. 2b1). The merged image shows that the IL-8 expression is mainly present in GFAP positive tumor cells, preferentially in the perinecrotic region (Fig. 2b8). An image of another perinecrotic area was taken at different focal distances (z-stacking) (Fig. 2b9, white box). In the Z-stacked image, robust IL-8 expression was observed largely in the cytoplasm of GFAP positive cells that are in direct contact with necrotic tissues (Fig. 2b10, merged in pink).

### Necrotic cells induce IL-8 expression and cell invasion

Incubation with necrotic cells induced both cell migration and IL-8 expression in human glioblastoma cells (Fig. 1); however, we asked whether these two functional outcomes of necrotic cell incubation occurred in identical or different sets of cells. To address this question, we performed a spheroid invasion assay with CRT-MG cells stably transfected with construct IL-8p-d2EGFP, which is a destabilized enhanced green fluorescent protein (EGFP)-expressing plasmid under the control of the IL-8 promoter. Multicellular tumor spheroids generated with the stable transfectants, CRT-MG/IL-8p-d2EGFP, were treated with necrotic cells for 1–4 days and then invading cells were analyzed by fluorescence microscopy, as described in the Methods section. A low level of basal expression of IL-8 was observed in the unstimulated control, and this expression was strongly enhanced by incubation with necrotic cells. At the same time, incubation with necrotic cells increased the invasion area of CRT-MG/IL-8p-d2EGFP (Fig. 2c, right panel). These results indicate that both functional outcomes of necrotic cell incubation, cell invasion and IL-8 expression, occur together in human glioblastoma cells.

### Necrotic cells induce AP-1 and NF-κB activation and DNA binding to the IL-8 promoter

**Figure 3:**
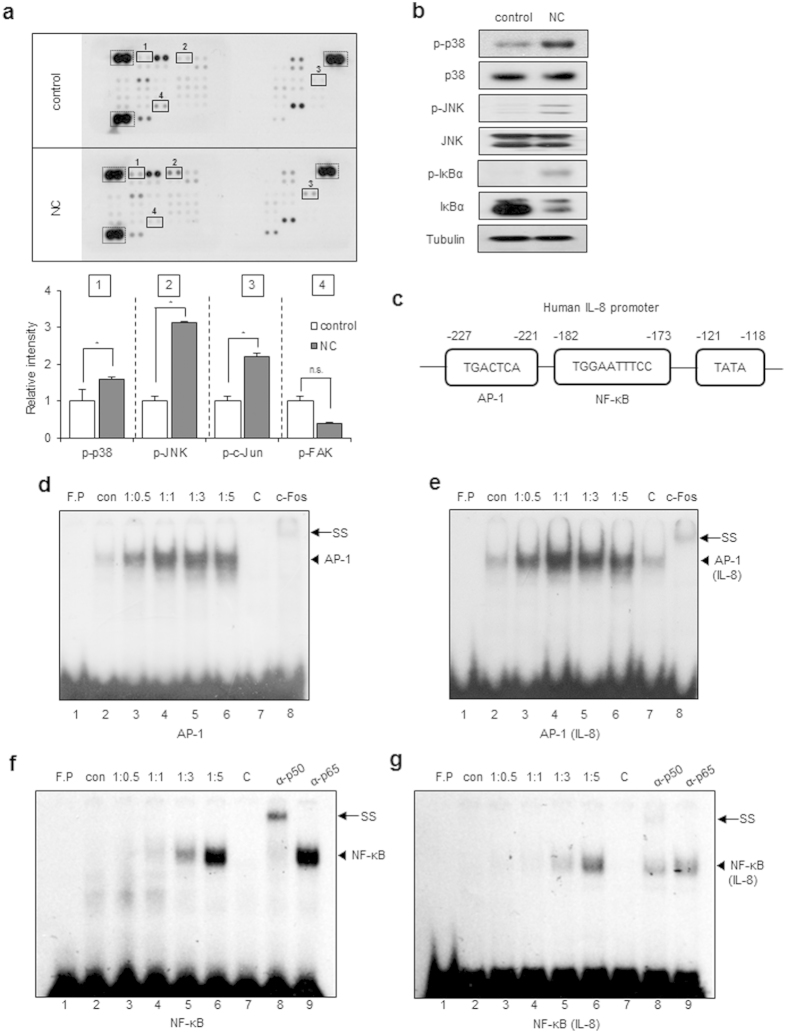
Necrotic cells induce phosphorylation of p38, JNK, and IκBα and IL-8 induction. (**a**) CRT-MG cells were either untreated (control) or treated with necrotic cells (NC) for 24 h, and 430 μg of cell lysate were used for the phosphokinase assay as indicated in Methods. The duplicate spots corresponding to the increased phosphorylation of p38, JNK, and c-Jun and decreased phosphorylation of FAK are highlighted in the solid box (left). The dashed line box indicates reference spots. The intensity of spots corresponding to p38, JNK, c-Jun, FAK, and the positive control was quantified using Image J software and subtracted from the background, then expressed as a ratio to the positive control. *P < 0.05 vs. each control. n.s., not significant. (**b**) Cell lysates from the CRT-MG cells treated with necrotic cells for 24 h were analyzed by immunoblotting to confirm the phosphokinase array results. Tubulin was used as the loading control. (**c**) The human IL-8 promoter AP-1 and NF-κB binding elements are indicated. (**d**–**g**) The nuclear extracts (NEs) from CRT-MG cells treated with or without necrotic cells were incubated with a radiolabeled DNA probe for the human IL-8 promoter and consensus oligonucleotides and subjected to an electro mobility shift assay. NEs were analyzed with probe for either AP-1 consensus oligonucleotide (**d**), AP-1 sequence within the IL-8 promoter (**e**), NF-κB consensus oligonucleotide (**f**), or NF-κB sequence within the IL-8 promoter (**g**). The competition assay was performed by adding a 100-fold molar excess of cold probe. Anti-c-Fos, p50 or -p65 antibodies were added to test the specificity of interaction. Data shown represent at least three experiments. F.P, free probe; C, competition; SS, supershift.

To investigate the signal transduction pathways that are activated in glioblastoma cells in response to necrotic cells, cell lysates obtained from untreated or necrotic cell-treated CRT-MG cells were analyzed using a phosphokinase signaling array. Compared to control array, the intensity of p38, JNK, and c-Jun duplicated spots in the arrays incubated with necrotic cells was significantly increased (Fig. 3a; highlighted in solid box; 1, 2, and 3), indicating increased levels of phosphorylation, whereas that of FAK was decreased, indicating decreased phosphorylation, but not significant (Fig. 3a; solid box; 4). To confirm these results, whole cell lysates from untreated or necrotic cells-treated CRT-MG cells, were analyzed by immunoblotting with phosphorylation specific antibodies for p38 and JNK (Fig. 3b). Consistent with the phosphokinase array data, phosphorylation of p38 and JNK was increased in necrotic cell-treated CRT-MG cells, compared to untreated control cells. Additionally, we analyzed IκBα phosphorylation and total IκBα expression levels. IκBα was slightly phosphorylated and total IκBα protein was reduced after being cultured with necrotic cells, indicating that necrotic cells activate the NF-κB pathway, as well as the AP-1 (c-Jun/c-Fos) pathway. The IL-8 promoter contains potential binding sites for transcription factors such as AP-1 and NF-κB^20^. A diagram of the transcription binding sites in the human IL-8 gene is shown in Fig. 3c. To test whether activated c-Jun and NF-κB bound to the IL-8 promoter, nuclear extracts (NEs) from untreated or different ratios of necrotic cells-treated CRT-MG cells were subjected to an electrophoretic mobility shift assay (EMSA). AP-1 DNA binding to the AP-1 consensus oligonucleotide was induced by 1:0.5 of necrotic cell treatment (lane 3, Fig. 3d) and remained high through 1:3 (lanes 4–5). The same NEs were exposed to a radiolabeled DNA probe containing the putative AP-1 binding element of the IL-8 promoter. AP-1 DNA binding to the IL-8 promoter was weakly induced in NE from 1:0.5 and 1:1 necrotic cell treated cells (lanes 3–4, Fig. 3e). Competition using a 100-molar excess unlabeled oligonucleotide abrogated complex formation (lane 7, Fig. 3d,e). Anti-c-Fos antibody supershifted the complex (lane 8). NF-κB DNA binding to the consensus oligonucleotide was observed in 1:1 necrotic cell-treated CRT-MG cells (lane 4, Fig. 3f), and binding activity was largely enhanced in 1:3 and 1:5 necrotic cell treated groups (lane 5–6). Utilizing a DNA probe containing the putative NF-κB binding element of the IL-8 promoter, binding was induced in necrotic cell-treated CRT-MG cells in ratio-dependent manner (Fig. 3g). Competition with excess unlabeled NF-κB oligonucleotide abrogated complex formation (lane 7, Fig. 3f,g). Anti-p50 antibody supershifted the complex (lane 8), whereas anti-p65 antibody did not (lane 9), confirming the specific interaction between p50 subunit and the NF-κB element. These results indicate that necrotic cells induce the activation of AP-1 and NF-κB signaling pathways, leading to enhanced DNA binding to the IL-8 promoter.

### NF-κB, JNK, and p38 inhibitors reduce IL-8 expression and cell migration and invasion in necrotic cell-treated CRT-MG cells

**Figure 4:**
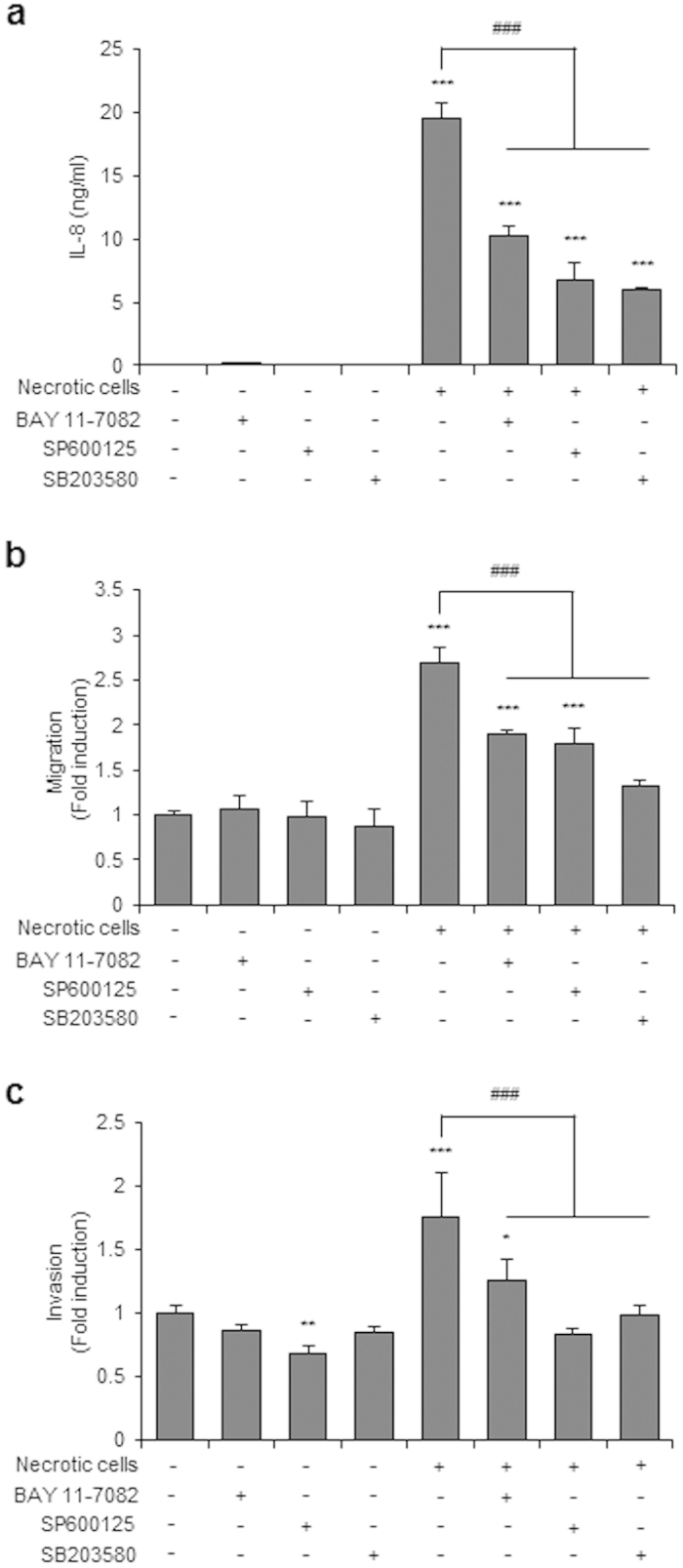
NF-κB, JNK, and p38 inhibitors reduced IL-8 expression, cell migration, and invasion in CRT-MG cells. CRT-MG cells were pretreated with an NF-κB inhibitor (BAY 11-7082 [BAY]), a JNK inhibitor (SP600125 [SP]), or a p38 inhibitor (SB203580 [SB]) for 30 min and then exposed to necrotic cells for 24 h. (**a**) Indicated supernatants from each conditions were collected, and IL-8 protein levels were measured by ELISA. The data represent three independent experiments. (**b**) Scratch wound healing assay in CRT-MG cells treated with necrotic CRT-MG cells and inhibitors incubated for 24 h. Migration activity was measured by calculating the area that advanced from boundary lines of the scratch to the cell-free space. Data are presented as the fold induction compared with each untreated control cells. (**c**) Invasion of CRT-MG cells was analyzed with a spheroid invasion assay. Spheroids were grown in the presence or absence of necrotic cells and inhibitors. Cell invasion was quantitated by calculating the invading area as indicated in Methods. The extent of invasion in the untreated control was set as 1; data are presented as the fold induction compared with untreated control cells. These parameters were analyzed in seven independent spheroids per condition. *P < 0.05, **P < 0.01, ***P < 0.001 vs. control, ^*###*^*P* < 0.001 vs. necrotic cells.

Necrotic cell-treated CRT-MG cells showed enhanced JNK and p38 phosphorylation and phosphorylation and degradation of IκBα (Fig. 3b). Therefore, we next examined the contribution of NF-κB, JNK, and p38 to necrotic cell-induced IL-8 expression, migration and invasion in glioblastoma cells, using specific inhibitors for NF-κB, JNK, and p38: BAY 11-7082 for NF-κB, SP600125 for JNK, and SB203580 for p38. Inhibition of the NF-κB and AP-1 signaling pathways suppressed both IL-8 expression and cell migration in necrotic cell-treated CRT-MG cells (Fig. 4a,b). Furthermore, the spheroid invasion assay showed that the invasion of CRT-MG/IL-8p-d2EGFP cells treated with necrotic cells was most strongly inhibited by the JNK inhibitor SP600125 (Fig. 4c). These results collectively indicate that necrotic cells induce IL-8 expression through the NF-κB and AP-1 signaling pathways and influence glioblastoma migration and invasion.

## Discussion

**Figure 5:**
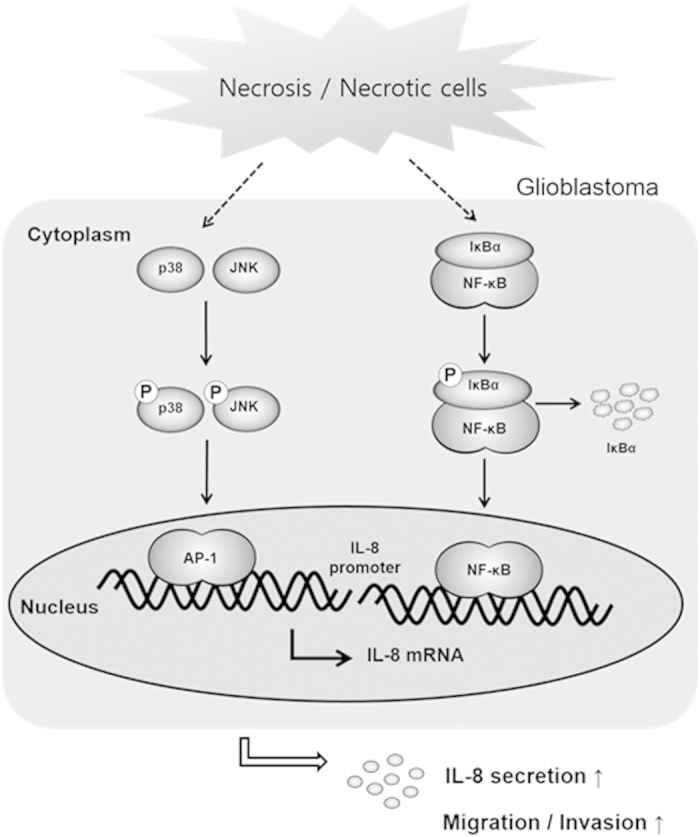
Proposed model for the effect of necrosis/necrotic cells in regulating glioblastoma invasion. Exposure to necrotic cells induces activation of NF-κB and AP-1 via phosphorylation of p38, JNK and IκBα and their binding to the IL-8 promoter, leading to increased IL-8 production and secretion, migration and invasion of glioblastoma cells.

In this study, we demonstrate that necrotic tissue influences glioblastoma migration through regulation of CXC chemokine IL-8 expression. Using the human GBM cell line CRT-MG and human GBM tissues, we found that incubation with necrotic cells or exposure to necrosis selectively induced the expression of IL-8 in glioblastoma cells. Necrotic cells induced phosphorylation of p38, JNK, and IκBα and binding of the transcription factors AP-1 and NF-κB to the IL-8 promoter, leading to increased IL-8 mRNA expression and IL-8 protein secretion (Fig. 5). Furthermore, NF-κB, JNK, and p38 inhibitors significantly inhibited IL-8 expression and the migration and invasion of CRT-MG cells induced by necrotic cells.

Induction of necrosis to kill cancer cell is one therapeutic goal for cancer treatment for several solid tumors^21^. However, in the case of GBM, the existence of necrosis and the survival of patients have an inverse correlation^18^. Therefore, we investigated the effect of necrotic cells on cell migration, intracellular signaling cascades, and chemokine expression in glioblastoma cells. To our knowledge, this is the first report to address the effect of necrotic cells on chemokine expression and migration of glioblastoma cells. In THP-1 human monocyte cells, pro-inflammatory cytokines such as TNF-α, IL-6, and IL-8 are released when cells are incubated with necrotic cell lysates^22^, similar to our results. Necrotic cell death, a characteristic feature of advanced solid tumors, results in the release of damage associated molecular patterns (DAMPs) such as HMGB1, calreticulin, and heat shock protein 90 (HSP90), into the extracellular space via the damaged plasma membrane^17^,^23^. Since necrotic cells contain numerous molecules including DAMPs, they have the potential to activate multiple signaling pathways. Surprisingly, we observed that fewer kinases were activated and phosphorylated in the phosphokinase array than expected (Fig. 3a). Of more than 500 human kinase, the phosphokinase array tests for just 43 kinases; thus, the potential contribution of other kinases cannot be fully excluded. However, our results obtained from the phosphokinase array suggest that, at least in CRT-MG cells, necrotic cells specifically activate the NF-κB and AP-1 signaling pathways.

IL-8 is a significant angiogenic, pro-inflammatory, and autocrine growth factors, and leads to a more invasive phenotype in cancer^10^,^11^,^12^,^13^,^14^. Tumor-associated macrophages secrete IL-8, promoting cell proliferation, invasion, and migration in cancer cells^7^. In glioblastoma, secreted IL-8 mediates cancer cell invasion and recapitulated angiogenic features, including ERK activation, tubulogenesis, and increased permeability^4^,^24^. Previous studies showed that IL-8 is induced in response to various stimuli, and the molecular mechanisms involved in IL-8 expression are well defined. Astrocytes and glioblastoma cells secrete IL-8 in response to LPS, IL-1β, TNF-α, ischemia, and hypoxia^25^,^26^,^27^. Although several factors are already known to be strong stimulators of IL-8, whether necrosis or necrotic cells trigger IL-8 production and induce cell migration, proliferation and neo-vascularization was unknown. We observed that recombinant IL-8 induces the migration of CRT-MG cells in a dose-dependent manner, consistent with previous studies (data not shown). Moreover, addition of neutralizing IL-8 antibody to necrotic cells significantly reduced migration in glioblastoma cells (Fig. 1c). Proliferation of CRT-MG cells was also increased by exposure to necrotic cells; however, the inhibitory effect of IL-8 neutralizing antibody on cell proliferation was not observed (Supplementary Fig. S3). The reason for the different response of IL-8 neutralization may be related to the factors involved in cell migration and proliferation of glioblastoma in necrotic microenvironment. Our results suggest the possibility that proliferation of glioblastoma in response to necrotic cells occurs by different necrotic cell-derived factors independently of IL-8. Previous studies have shown that IL-8 expression is localized in perivascular and oxygen deprived cells surrounding necrotic regions^14^,^28^. In our system, IHC data showed that IL-8 induction is found mainly at the edge of the necrotic zone, similar to previous reports. Our results clearly demonstrate that necrotic cells increase IL-8 production and cell migration and invasion in glioblastoma. Utilizing human glioblastoma cell lines and human tissues, significant expression of IL-8 in response to necrotic cells/necrosis was observed. IL-8 promotes tumoral angiogenesis and increases brain endothelial cell permeability in glioblastoma^14^,^24^. Therefore, it will be interesting to compare both direct and indirect effect of necrotic cells on neo-vascularization and to determine whether glioblastoma cell-derived IL-8 mediates the process of angiogenesis, similar to cell migration and invasion.

The genomic IL-8 sequence includes a 1.5-kb 5′-flanking region that contains several potential binding sites for known transcription factors, such as AP-1, NF-IL6, and NF-κB^29^. In the human pancreatic tumor cell line, NF-κB regulates the expression of c-Fos and the activity of AP-1 in response to ROS and serum condition^30^. In CRT-MG cells, caffeic acid phenethyl ester (CAPE) abrogated TNF-α-induced expression of chemokine (C-C motif) ligand 2 (CCL2) and intercellular adhesion molecule 1 (ICAM-1) via inhibition of NF-κB activation, while increased TNF-α-induced CXCL-8 expression through JNK and AP-1 activation^31^. In another GBM cell line, U251-MG, IL-8 mRNA and protein expression were increased by Ca^2+^-ionophore and phorbol-myristate-acetate via NF-κB and AP-1 signaling, contributing to invasive potential^32^. Our results showed that necrotic cells induced p38, JNK, c-Jun, and IκBα phosphorylation (Fig. 3a,b), leading to increased IL-8 transcription and expression, by enhanced DNA binding of NF-κB and AP-1 to the IL-8 promoter. Interestingly, AP-1 DNA binding was weakly induced in 1:0.5 and 1:1 necrotic cell-treated cells, and NF-κB binding activity was largely enhanced in 1:3 and 1:5 necrotic cell-treated groups. In previous studies, it was shown that the kinetics of stimulation of NF-κB differ from those of AP-1 in response to dopamine (DA); AP-1 is activated at 8 h and activation of NF-κB appears at 24 h^33^. Together with our data, this suggests that activation of NF-κB and AP-1 may be differentially regulated in response to the same stimuli as per varied time point or varied concentration. Our results suggest that necrotic cells activate both AP-1 and NF-κB, and switch the key transcription factor from AP-1 to NF-κB in response to the differential ratio of necrotic cells. The biological significance is unclear, but IL-8 expression levels might be affected by the extent of necrosis. Furthermore, this suggests that a drastic increase in IL-8 production induced by large-scale necrosis is mediated mainly by NF-κB. Again, since we observed that NF-κB, JNK, and p38 inhibitors significantly inhibit necrotic cell-induced IL-8 expression and the migration and invasion of CRT-MG cells (Fig. 4), our results indicate that necrotic cells increase the invasive phenotype of GBM cells through activation of NF-κB and AP-1 signaling pathways.

Our results demonstrate that, in glioblastoma, necrotic tissues are significant regulators of glioblastoma migration and invasion. In the current study, we aimed to investigate the effect of necrosis on cell migration, invasion, intracellular signaling cascades, and chemokine expression in glioblastoma cells. Further studies are needed to identify factors within necrotic cells that trigger these responses and the target receptors in glioblastoma cells that mediate such responses. Herein, we demonstrate the underlying mechanisms of GBM metastasis in the presence of necrotic cells and identify the signaling pathways in cancer cells that are activated by necrotic cells, providing a better understanding of the effect of necrosis on glioblastoma.

## Methods

### Cells

Human glioblastoma CRT-MG, U251-MG and U87-MG cells were maintained in Dulbecco’s modified essential medium (DMEM, WelGENE Inc. Daegu, Korea) containing 10% fetal bovine serum (FBS, Gibco, Grand Island, USA), L-glutamine, 100 U/ml penicillin, and 10 μg/ml streptomycin. Stable cell line CRT-MG/IL-8p-d2EGFP cells were kindly provide by Dr. Chulhee Choi (KAIST, Daejeon, Korea) and maintained as previously described^34^. For preparation of necrotic CRT-MG cells were frozen and thawed through five cycles of liquid nitrogen-water bath treatment^35^.

### Glioblastoma multiform (GBM) tissues

Twelve cases of human glioblastoma tissue specimens were selected from the department of pathology, Ewha Womans University Mokdong hospital, over the past 3 years (2012–2014). Hematoxylin-eosin-stained slides were reviewed and all cases showed variable degree of geographic necrosis. This study protocol was approved by the Institutional Review Board (IRB) of the Ewha Womans University Mokdong hospital (IRB protocol number: EUMC 2016-01-005). Informed consent had been obtained from each patient prior to surgery and was waived in this study by IRB due to the retrospective nature. All experiments were performed in accordance with relevant guidelines and regulations.

### Reagents and antibodies

Polyclonal anti-human IL-8 was obtained from R&D Systems (Minneapolis, MN, USA). IgG from goat serum was purchased from Sigma-Aldrich, Co. (St. Louis, MO, USA). Antibody to anti-p-JNK, anti-p-p38, anti-p38 and anti-IκB-α antibody were purchased from Santa Cruz Biotechnology (Santa Cruz, CA, USA) and anti-JNK, anti-p-IκB-α (ser32) was purchased from Cell Signaling Technology (Beverly, MA, USA). Anti-tubulin antibody was purchased from Sigma-Aldrich, Co. Horseradish peroxidase (HRP)-conjugated secondary antibodies for immunoblotting were obtained from Santa Cruz Biotechnology. The NF-κB inhibitor BAY 11-7092, JNK inhibitor SP600125, p38 inhibitor SB203580 were purchased from Sigma-Aldrich, Co.

### Scratch wound healing assay

CRT-MG cells were scraped off from the bottom of a culture plate using a pipette tip to create a cell-free area as previously described^36^. CRT-MG cells were washed with DMEM to remove cell debris and then incubated with necrotic cells for 24 and 48 h in 1% FBS culture media. The wound area was photographed after scratching for control. Migration activity was measured by the area that advanced from 0 h control to cell-free space at the intervals of 24 h–48 h after scratching^37^, and we quantified using Image J software (developed by Wayne Rasband, National Institutes of Health, Bethesda, MD; available at (http://rsb.info.nih.gov/ij/index.html).

### Human chemokine array

Cytokines, chemokines and angiogenic factors using commercially available protein array systems (Proteome profiler^TM^ Arrays, R&D Systems) according to the manufacturer’s protocol. CRT-MG cells were either untreated or treated with necrotic cells for 24 h, and then supernatants were collected and subjected to protein array. Sample and reconstituted detection antibody mixture were incubated at RT for 1 h and the prepared mixtures were added on each membrane at 4 °C for overnight. And diluted streptavidin-HRP treated into each membrane for 30 min at RT on a rocking platform shaker. Membranes were exposed to blue X-ray film (AGFA, Mortsel, Belgium) by using chemiluminescent reagent for 3–10 min. The changed spot intensities were measured by the spot intensity divided by reference spot intensity, which using Image analysis software.

### Enzyme-linked immunosorbent assay (ELISA)

Chemokine levels were measured in the culture supernatant of CRT-MG seeded in 60 mm culture plates. After stabilization cell, were treated with or without necrotic CRT-MG cells for 24 h. Supernatants were collected and centrifuged at 12,000 rpm for 20 min and chemokine concentrations were determined by a sandwich ELISA method with IL-8 (BioLegend, San Diego, CA, USA) following the procedure provided by the manufacturer. The concentration of chemokine in each sample was determined with reference to a standard curve generated by known amounts of IL-8.

### Quantitative polymerase chain reaction (qRT-PCR)

Total RNA isolated using RNA extract kit (easy-Blue^TM^, Intron, Gyeonggido, Korea). cDNA was synthesized from total RNA and qRT-PCR was performed on an ABI StepOnePlus^TM^ Real-Time PCR machine (Applied Biosystems, Foster City, CA, USA) using a Power SYBRH Green PCR Master Mix (Applied Biosystems) according to the manufacturer’s protocol. The primer sequences were as follows: IL-8, forward 5′-CTCCAAACCTTTCCACCCC-3′, reverse 5′-GATTCTTGGATACCACAGAGAATG-3′; GAPDH, forward 5′-TGGAAATCCCATCACCATCT-3′, reverse 5′-GTCTTCTGGGTGGCAGTGAT-3′. Quantitation approach was performed by termed the comparative Ct method as previously described^38^. The Ct values of both the control and the samples of interest are normalized to an appropriate GAPDH gene (ΔCt = IL-8 Ct – GAPDH Ct). Relative expression levels were calculated as 2^−ΔΔCt^ method, where ΔΔCt = IL-8 sample ΔCt sample – IL-8 control ΔCt.

### Immunohistochemistry and immunofluorescence analysis for IL-8

Four μm-sections were taken from formalin-fixed, paraffin-embedded tissue blocks used for immunohistochemistry. Immunohistochemical staining was done using automated machine (Bond™ Automated Immunohistochemistry, Leica, Wetzlar, Germany) and the Bond polymer detection system with counterstain (Leica). Slides were briefly deparaffinized in xylene twice for 5 min and then rehydrated with graded ethanol solutions and distilled water. Antigen was than retrieved by heating the sections in 10 mM citrate buffer (pH 6.0) for 20 min. Then endogenous peroxidase was blocked by 3% hydrogen peroxide for 5 min. Slides were incubated with primary antibody for IL-8 (1/400 dilution) at RT for 30 min, mouse anti-goat secondary antibody (1/100 dilution) for 3 min, and polymeric HRP-linker antibody conjugates for 5 min. 3,3′-diaminobenzidine was used for detection. For immunofluorescence analysis, GBM tissue slides were stained with anti-IL-8 for 1 h, and then stained with anti-goat Alexa fluor 568 conjugated (Molecular Probes, Eugene, OR, USA) for 90 min. Cells were rinsed again with PBS, followed by staining with mouse anti-GFAP antibody for 1 h and incubated with anti-mouse Alexa fluor 488 conjugated secondary antibodies (Molecular Probes) for 90 min. Tissues were mounted in mounting solution containing DAPI (Vector Laboratories, Inc., Burlingame, USA) and observed using confocal microscope (Olympus, Melville, NY, USA).

### Spheroid invasion assay

Multicellular tumor spheroids were generated by using the hanging-drop method^39^. In brief, CRT-MG/IL-8p-dEGFP cells were detached with EDTA (2 mM), resuspended in RPMI1640 with 10% FBS, 20% methylcellulose (Sigma-Aldrich, Co.), and 1% Matrigel (BD Biosciences, San Jose, California), and incubated as droplets (25 μl) containing 1,000 cells for 48 h to ensure multicellular aggregation. For collagen invasion assays, spheroids were mixed with rat-tail collagen solution (3 mg/ml, BD Biosciences), pipetted as a drop-matrix, and then polymerized at 37 °C. Necrotic cells were added to the spheroid cultures 24 h after seeding. The invasion area was measured by using Image J software as previously described with minor modification^40^. In brief, invading area was calculated as follows: total area minus spheroid body area. These value were analyzed in seven independent spheroids per condition. The experiment was performed in triplicates.

### Human phosphokinase array

Cells were treated with or without necrotic cells for 24 h, and then lysed with array lysis buffer. Quantitation of protein concentration was performed using BCA assay. Cell lysates (430 μg of total proteins per array) were applied to the phosphoprotein array following the manufacturer’s protocol (Proteome Profiler^TM^ Arrays, R&D Systems). Block buffer was contacted into each membrane for 1 h on a rocking platform shaker. Cell lysates were diluted in array buffer and were incubated overnight at 4 °C. The array was incubated in each reconstituted detection antibody cocktail for 2 h at RT, then was diluted streptavidin-HRP for 30 min, followed by application of chemiluminescent reagent and expose to X-ray film (AGFA). The phosphokinase activity was measured by the changed spot intensity divided by reference spot intensity, which using Image J software.

### Western blot analysis

Cells were treated with or without necrotic cells for 24 h, and lysed in RIPA buffer containing Xpert Protease Inhibitor cocktail (GenDEPOT, Inc., Baker, TX, USA). Lysates were centrifuged (12,000 rpm) at 4 °C for 30 min and 20 μg of protein was subjected to 10% SDS/PAGE, and then blots were probe with p-p38, p38, p-JNK, JNK, p-IκB-α, IκB-α, and tubulin aticodies. After incubation with specific secondary antibodies, blots were developed using ECL chemiluminescence system (Amersham, Buckinghamshire, UK) and exposed to blue X-ray film (AGFA).

### Nuclear extracts and electrophoretic mobility shift assays (EMSA)

EMSA was performed with 3 μg of nuclear extracts, as previously described^36^. In brief, nuclear extracts from CRT-MG treated with or without necrotic cells were incubated with consensus oligonucleotides or the IL-8 sequence including putative NF-κB or AP-1 binding element, which was end-labeled with [^32^P] ATP using T4 polynucleotide kinase. Bound and free DNA were resolved by electrophoresis in 5% polyacrylamide gels using 0.5X TBE as running buffer and assessed by autography. For competition experiments, a 100-molar excess of unlabeled oligonucleotide was added to the nuclear extracts for 10 min before addition of the labeled probe. For supershift experiments, binding reactions containing nuclear extract were incubated for 30 min at RT with c-Fos, anti-p50 or anti-p65 antibody (Santa Cruz). The oligonucleotide probes used were as follows: NF-κB consensus, forward 5′-AGTTGAGGGGACTTTCCCAGGC-3′; NF-κB-IL-8, forward 5′-ATCGTGGAATTTCCTCTGACAT-3′; AP-1 consensus, forward 5′-CGCTTGATGACTCAGCCGGAA-3′; AP-1-IL-8, forward 5′-GATGACTCAGGTTTGCCCTGAG-3′.

### Flow cytometry

The proportion of apoptosis and necrosis was examined using BD Pharmingen^TM^ FITC Annexin V Apoptosis detection Kit I (BD Biosciences) according to the manufacturer’s protocol. Necrotic cells were stained with AnnexinV-fluorescence isothiocyanate (FITC) and propidium iodine (PI) in the dark at RT for 15 min. Cells were analyzed by FACSCalibur™ flow cytometry (BD Bioscience). Necrotic cells were defined as PI-positive and Annexin V-FITC negative.

### Cell proliferation assay

Cell proliferation was assessed using WST-8-[2-(2-methoxy 4-nitrophenyl)-5-(2,4-disulfophenyl)-2H-tetrazolium monosodium salt] (Cell Counting Kit 8 (CCK-8); Dojindo molecular technology, Inc., Kumamoto, Japan). CRT-MG cells (1 × 10^4^) were seeded into 96 well culture plates and treated with necrotic cells for 24 and 48 h. CCK-8 solution (1/10 vol/vol) was added to each well, and the plates were incubated for 3 h at 37 °C. Finally, absorbance at 450 nm was determined using a microplate reader (VERSA max microplate reader, Molecular Devices, Orleans, USA).

### Statistical analysis

Mean ± standard error of the mean (SEM) were showed in all bar graphs from independent experiments, and statistical significance was analyzed by ANOVA and student t-test using the SPSS software version 23.0 (SPSS Inc., Chicago, IL, USA). Statistical significance of the data was set a value of *p* < 0.05.

## Additional Information

**How to cite this article**: Ahn, S.-H. *et al.* Necrotic cells influence migration and invasion of glioblastoma via NF-κB/AP-1-mediated IL-8 regulation. *Sci. Rep.* **6**, 24552; doi: 10.1038/srep24552 (2016).

## Supplementary Material

Supplementary Information
