# Supplementary material for: Necrotic cells influence migration and invasion of glioblastoma via NF-κB/AP-1-mediated IL-8 regulation
Source: Sci Rep. 2016 Apr 14;6:24552. doi: 10.1038/srep24552 (PMC4830983; doi:10.1038/srep24552)

Supplementary Information

Necrotic cells influence migration and invasion of glioblastoma via NF-κB/AP-1-mediated IL-8 regulation

So-Hee Ahn[1, 2], Hyunju Park[1, 2], Young-Ho Ahn[2, 3], Sewha Kim[4], Min-Sun Cho[4], Jihee Lee Kang[1, 2], Youn-Hee Choi[1,2*]

[1]Department of Physiology, [2]Tissue Injury Defense Research Center, [3]Department of Molecular Medicine, [4]Department of Pathology Ewha Womans University School of Medicine, Seoul 911-1, Korea.

* Correspondence to Youn-Hee Choi, M.D., Ph.D., Department of Physiology, School of Medicine, Ewha Womans University, 911-1 Mok-dong Yangcheon-gu, Seoul, 158-710, Korea. Phone: +82-2-2650-5838, Fax: +82-2-2650-5717, E-mail: yc@ewha.ac.kr

Supplementary Figure S1. Preparation of necrotic cells. CRT-MG cells were frozen and thawed through five cycles of liquid nitrogen-water bath. Cells were stained using Annexin Ⅴ -FITC and propidium iodide (PI), and then the quantitation of apoptosis/necrosis was determined by flow cytometry. The proportion of necrotic cells stained positive for PI was seen to consist of over 90% of prepared cells. NC, necrotic cells.

Supplementary Figure S2. Necrotic cells increase migration and IL-8 expression in U251-MG and U87-MG cells. (a,b) U251-MG and U87-MG cells were treated with necrotic cells for 0, 24 and 48 h. Migration activity was measured by calculating the area that advanced from boundary lines of scratch to cell-free space for 24 or 48 h. Data are presented as the fold induction compared with each untreated control cells. *$P < 0.05$, **$P < 0.01$, ***$P < 0.001$ vs. control. (c-e) CRT-MG, U251-MG and U87-MG cells were either untreated or treated with different ratios of necrotic cells (NC) for 24 h as indicated. After incubation, supernatants from each condition were collected and IL-8 protein levels were measured by ELISA. Data were presented as fold induction compared with control cells. *$P < 0.05$, **$P < 0.01$, ***$P < 0.001$ vs. control.

Supplementary Figure S3. Necrotic cells affect proliferation of CRT-MG cells. (a) CRT-MG cells (1×10^4) were seeded and treated with necrotic CRT-MG cells for 24 h. CCK-8 solution was further incubated for 4 h and absorbance was measured at 450 nm using a microplate reader to cell proliferation. ***$P < 0.001$ *vs.* control. (b) CRT-MG cells treated with necrotic cells in the presence or absence of either neutralizing IL-8 antibody (2.5 μg/ml) or control IgG (2.5 μg/ml) for 24h. Proliferation was measured by CCK-8. ***$P < 0.001$ *vs.* control. n.s., not significant. Data shown are representative of at least three experiments.

Supplementary Fig. S1

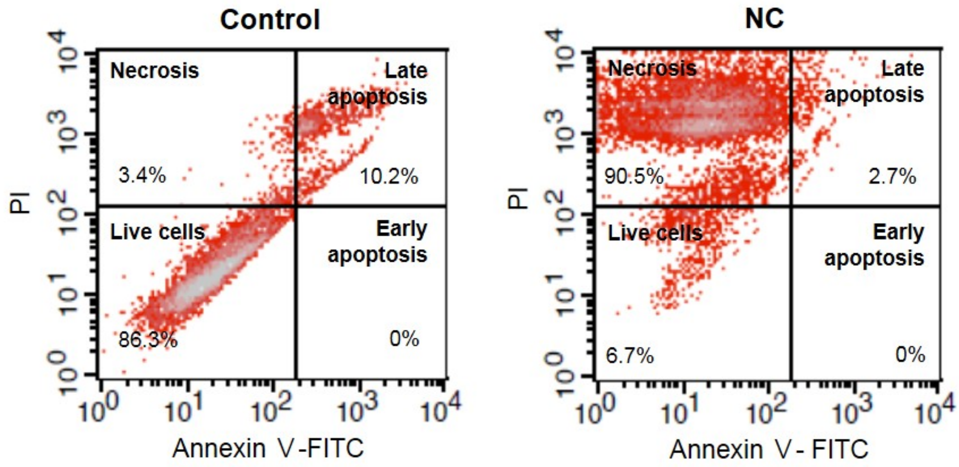

Supplementary Fig. S2

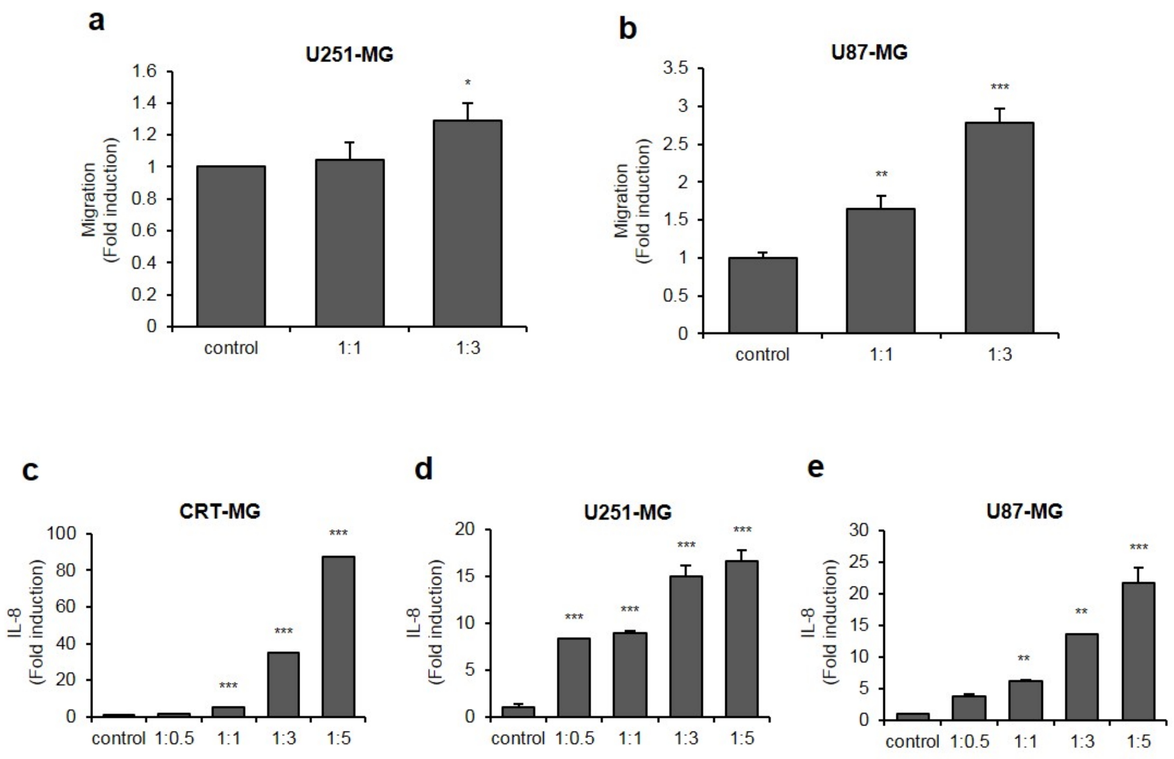

Supplementary Fig. S3

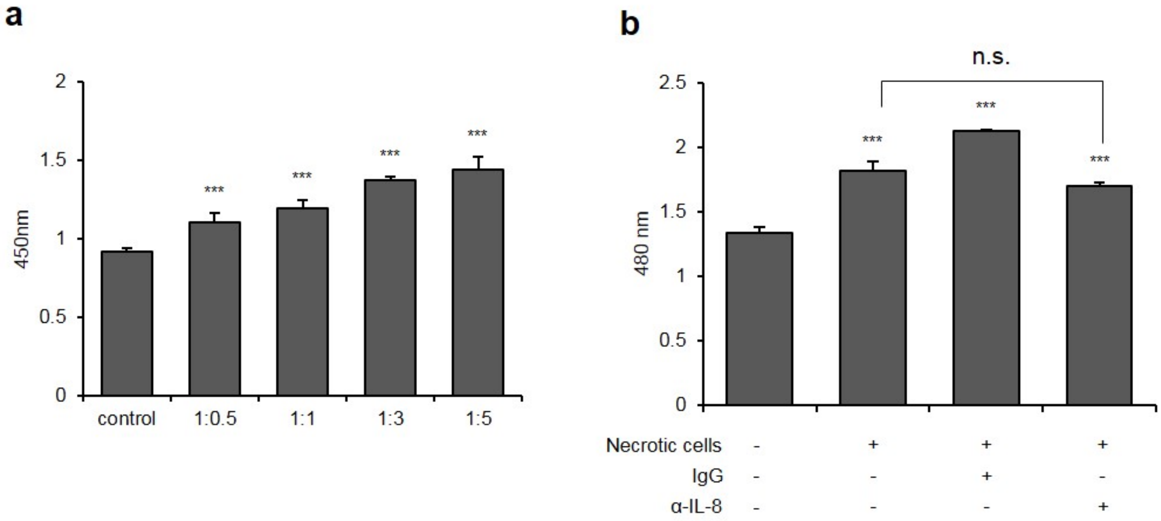

Supplement: Supplementary Information [file srep24552-s1.pdf]
